# Obesity, gestational weight gain, and polyunsaturated fatty acids profile in pregnant Saudi women

**DOI:** 10.6026/97320630016493

**Published:** 2020-06-30

**Authors:** Hala Al-Otaibi, Nahed Hussein, Hani Mustafa, Norah Al-Mudaires

**Affiliations:** 1 Department of Food Sciences and Nutrition, College of Agriculture and Food Sciences, King Faisal University, Al-Ahsa, Saudi Arabia; 2 Faculty of Specific Education, Ain Shams University, Cairo, Egypt; 3 King Abdul Aziz Hospital, National Guard Health Affairs, Al-Ahsa, Saudi Arabia; 4 Department of Physical Education, College of Education, King Faisal University, Al-Ahsa, Saudi Arabia

**Keywords:** Obesity, Omega-3 PUFAs, Pre-pregnancy BMI, Omega-6 PUFAs

## Abstract

Obesity and excessive gestational weight gain (GWG) are associated with a deficiency of essential fatty acids, affecting maternal health during and after pregnancy. Therefore, it is of interest to identify the associations of pre-pregnancy body mass index (BMI) and GWG with lipid profiles in Saudi women after giving birth. Hence, a cross-sectional study of 238 pregnant women aged 20-40 years was conducted at the King Abdul Aziz Hospital, in Al-Ahsa Governorate-Saudi Arabia. Thus, socio-demographic and anthropometric data were collected using a structured questionnaire. Poly-unsaturated fatty acids (PUFAs), saturated fatty acids (SFAs), and monounsaturated fatty acids (MUFAs) levels were assessed from blood samples collected after the women gave birth. The participants generally consumed diets low in omega-3 and omega-6 PUFAs and high in SFAs and MUFAs. Among them, 51% had university degrees, only 20.4% were employed, and 50% had pre-pregnancy overweight/obesity. Women with overweight/obesity had a higher omega-6 to omega-3 PUFA ratio than women with normal weight. Overweight, obesity, and excessive GWG were not associated with higher levels of total n-3 PUFAs, docosahexaenoic acid, and α-linolenic acid but were associated with higher levels of total n-6 PUFAs and linoleic acid. Women with obesity had significantly higher SFA and MUFA levels than women with normal weight, whereas women with excessive GWG were twice as likely to have higher SFA levels than women with adequate GWG. We show that a higher pre-pregnancy BMI and excessive GWG were significantly associated with abnormal lipid profiles in Saudi women after giving birth. We believe that future studies will help explore these associations in detail.

## Background

The rapid economic development and urbanization in Saudi Arabia have resulted in a lifestyle of high-sugar and high-fat diet and reduced physical activity, contributing to the rising incidence of overweight and obesity, especially among women [1]. According to the recent Riyadh Mother and Baby Multicenter Cohort Study (RAHMA), the prevalence of overweight and obesity in women in Saudi Arabia is 68.5% [2], which is considerably higher than that in Western countries (30%) and is the highest worldwide [3,4]. Obesity and excessive gestational weight gain (GWG) during pregnancy are the major risk factors for gestational diabetes, gestational hypertensive disorders, large-for-gestational-age birth weights, cesarean section, and high body fat percentage in infants that continues until adulthood [5].

During pregnancy, fatty acid (FA) levels rise progressively to increase the sources of metabolic and storage energy, reduces inflammation, and synthesizes prostaglandins [6]. Long-chain polyunsaturated fatty acids (PUFAs)-which are categorized into two main families, namely, omega-3 (n-3) PUFAs (e.g., docosahexaenoic acid [DHA]) and omega-6 (n-6) PUFAs (e.g., arachidonic acid [AA])-play a major role in fetal neural and retinal development, especially in the last trimester of pregnancy [7].

Adults who are overweight or obese usually have higher saturated fatty acid (SFA) levels, lower n-3 PUFA levels, and higher n-6 to n-3 PUFA ratios. [8] In pregnant women, obesity has been shown to be associated with lower plasma levels of DHA and AA [9]. Moreover, excessive GWG is associated with higher plasma levels of SFAs, monounsaturated fatty acids (MUFAs), and n-6 PUFAs and imbalances in n-6 to n-3 PUFA ratios [10].

There have been several studies conducted to investigate n-3 and n-6 PUFA levels among men [11] and women [12] and their associations with heart diseases. A recent study has shown that cardiovascular risk in the Saudi population is related to n-3 PUFA levels [13] and sickle cell disease [14]. To date,no studies in Saudi Arabia have compared FA levels between women with normal weight and women with overweight, obesity, or excessive GWG. This study therefore aimed to examine the associations of pre-pregnancy body mass index (BMI) and GWG with lipid profiles in Saudi women after giving birth.

## Materials and Methods:

### Study design and participants

This cross-sectional study included pregnant women aged 20-40 years from the Department of Obstetrics and Gynecology, King Abdul Aziz Hospital, Al-Ahsa, Saudi Arabia between Januarys to September 2018. Only healthy women with a singleton pregnancy and who delivered at term (total gestation ≥ 37 weeks) without medical or obstetric complications were included. Pregnant women with medical conditions such as multiple gestations or chronic diseases were excluded from the study.

**Figure 1:**
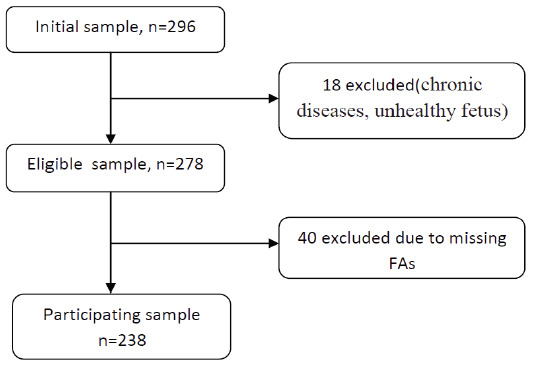
Workflow for the selection of participants in the study

The participants were given a brief explanation regarding the study objectives before providing written informed consent to participate in the study. A flowchart of the identificationed and screening of participants based on the eligibility criteria is shown in Figure 1. All procedures and protocols of this study were approved by the King Abdullah International Medical Research Center Ethical and Research Committee (No. RA17/021/A).

### Data collection

Data were collected using a structured questionnaire administered through face-to-face interview. The questionnaire included information about the following sociodemographic characteristics: age, education level, employment status, income, and maternal parity. Pre-pregnancy BMI was categorized according to the World Health Organization (WHO) standards [15]. GWG was computed by subtracting the pre-pregnancy weight from the weight at time of delivery, based on the pre-pregnancy BMI. The range in weight gain recommended for women with pre-pregnancy obesity, overweight, normal weight, and underweight was 5.0-9.0, 7.0-11.5, 11.5-16.0, and 12.5-18 kg, respectively. GWG was classified as inadequate, adequate, or excessive, based on the Institute of Medicine guidelines [16].

### Samples collection and FA analysis:

After delivery, 5 mL of venous blood following a 12 h overnight fasting was drawn from the participants into EDTA tubes. All blood samples were immediately centrifuged at 3000 rpm for 15 min to separate the plasma and erythrocytes. The plasma samples were stored at -80°C until further analysis.

For the analysis of FA levels, the plasma samples were transported to the Central Labs at the King Faisal University. FAs were extracted from the plasma and derivatized to FA methyl esters (FAMEs) using methods described elsewhere.17 The FAMEs were quantified and analyzed using gas chromatography-mass spectrometry (Shimadzu QP-2010 Plus, Kyoto, Japan). The FAs were analyzed in terms of percentage by weight of total FAs.

For our analyses, we selected FAs that were associated with the risk of cardiovascular diseases or pregnancy outcomes based on the findings of previous studies [9, 10]. The analyses included SFAs (stearic acid, C18:0; palmitic acid, C16:0; and myristic acid, C14:0), MUFAs (oleic acid, C18:1 n-9; and palmitoleic acid, C16:1 n-7), n-3 PUFAs (DHA, C22:6 n-3; and α-linolenic acid [ALA], C18:3 n-3), and n-6 PUFAs (AA, C20:4 n-6), dihomo-γ-linolenic acid (DGLA, C20:3 n-6), and linoleic acid (LA, C18:2 n-6).

### Statistical analysis

All data were presented as means and standard deviations or frequencies and percentages. For between-group comparisons, we used the one-way analysis of variance continuous variables and the chi-squared test for categorical variables. In the logistic regression model, the dependent variables were pre-pregnancy BMI category (reference: normal weight) and GWG (reference: adequate weight gain), and the independent variables were the FA levels. All statistical analyses were performed using SPSS version 23 (SPSS Inc., Chicago, IL, USA), and a P-value of <0.05 was considered as statistically significant.

## Results:

The study included a total of 238 pregnant women, of whom 118 (49.6%) had a normal weight, 86 (36.1%) were overweight, and 34 (14.3%) were obese. Table 1 summarizes the characteristics of pregnant women according to the BMI categories. Of all participants, 58.5% attained a bachelor’s degree and had a normal weight, and the difference was significant. The monthly income ranged from 6000 to 10,000 Saudi riyal, and most of the women were unemployed. Women with obesity had a significantly higher mean pre-pregnancy BMI (33.9 ± 4.1) (P < 0.001). Vaginal delivery was more common in women with normal weight (82.2%) and who were overweight (53.5%) than in those with obesity (20.6%; P < 0.001). The mean GWG was higher in women with obesity than in women with normal weight (79.4% vs. 5.1%), and the difference was significant.

Table 2 shows the maternal plasma FA profiles according to the BMI category. The percentage of n-3 PUFAs was substantially lower than that of n-6 PUFAs, yielding a high n-6 to n-3 PUFA ratio of 15.5 for obesity, 13.5 for overweight, and 9.4 for normal weight. The total SFA levels were significantly higher in women with obesity than in those with overweight and normal weight, while no significant difference was observed for total MUFA levels between all groups. In terms of levels of individual n-3 PUFAs, no significant difference was found between all groups, and among the n-6 PUFAs, only LA showed a significant difference between the groups.

Logistic regression analyses showed that women with obesity had higher levels of SFAs (odds ratio [OR], 1.01; 95% confidence interval [CI], 0.84-1.29) and MUFAs (OR, 0.83; 95% CI, 0.68-1.1) than women with normal weight, whereas women with excessive GWG were twice as more likely to have higher levels of SFAs (OR, 2.02; 95% CI, 0.81-1.07) than women with adequate GWG. The independent variables (overweight, obesity, inadequate GWG, and excessive GWG) were not associated with higher levels of total n-3 PUFAs, DHA, and ALA. However, overweight (OR, 1.101; 95% CI, 1.04-1.16), obesity (OR, 1.21; 95% CI, 1.11-1.31), and excessive GWG (OR, 1.06; 95% CI, 1-1.12) were significantly associated with higher total n-6 PUFA levels. Furthermore, overweight, obesity, and excessive GWG were significantly associated with higher LA levels (Table 3).

## Discussion:

Overweight and obesity are major public health problems worldwide. In Saudi Arabia, according to the WHO, 65.9% of females aged ≥ 15 years are overweight or obese [18]. Accumulating evidence from a recent meta-analysis revealed that maternal pre-pregnancy overweight, obesity, and excessive GWG are risk factors for overweight and obesity in children [19]. These conditions are also associated with exaggerated physiological insulin resistance, which causes excessive transport of glucose and free FAs (overnutrition) to the fetus, further leading to long-term negative metabolic programming [20]. In this study, the frequency of cesarean sections was higher in women with overweight and obesity than in women with normal weight, and this can be attributed to the presence of excessive fat in soft tissues that reduce the rate of cervical dilatation, hence the need for a cesarean section delivery. This finding has also been observed in previous studies [21,22].

In this study, 50% of the women were categorized as being overweight or obese, which is similar to the findings of previous studies conducted in Saudi Arabia [22, 23]. In contrast, the RAHMA study reported a higher prevalence, at 68.5%.2 This difference in findings could be owing to the fact that the participants in this study were more educated than those in the RAHMA study (58.5% vs. 41.6% of women with normal weight held a bachelor's degree). A regional study conducted in Qatar and Lebanon found that a higher educational level was associated with lower odds of pre-pregnancy obesity and overweight [24]. These findings suggest that well-educated women have more access to and are more aware of nutritional information that may contribute to improving their dietary and lifestyle behaviors and prevent them from being overweight or obese. In this study, almost 80% of women with obesity had excessive GWG compared with those with overweight (40.7%) and normal weight (5.1%). Similarly, other studies reported that women with overweight or obesity are more likely to experience excessive GWG [25,26].

Our study also revealed that pregnant women with overweight and obesity had higher levels of MUFAs, SFAs, total n-6 PUFAs, and LA and lower levels of total n-3 PUFAs, DHA, and ALA. Similar results have been reported by Vidakovic et al. [10] who found that a higher pre-pregnancy BMI was associated with higher levels of total SFAs and total n-6 PUFAs, whereas excessive GWG was associated with higher levels of total SFAs, MUFAs, and n-6 PUFAs. Likewise, Tomedi et al., [9] reported that American women who were pregnant and had normal weight had higher DHA and AA levels than those with overweight and obesity. Meanwhile, other studies concluded that women with high first-trimester weight gain had low levels of n-3 PUFAs in their plasma and that increased intakes of n-3 PUFAs may improve body composition [25, 27]. Furthermore, an increase in maternal n-6 PUFAs and a decrease in n-3 PUFAs might affect fetal growth, contributing to the intergenerational cycle of obesity, and have been reported to be associated with a higher BMI in children aged 4-7 years [28,29].

The food habits in Saudi Arabia are similar to those in Western countries-typically low in seafood and high in plant oils (source of n-6 PUFAs), causing an increase in the n-6 to n-3 PUFA ratio, which is associated with obesity and other health problems, such as nonalcoholic fatty liver disease [30]. Consistent with our results, previous studies in Western countries also reported that pregnant women with overweight and obesity had a higher n-6 to n-3 PUFA ratio than women with normal weight.10 Cinelli et al., [31] reported that pre-pregnancy overweight and obesity were associated with higher SFA and n-6 PUFA levels and lower LA levels and that excessive GWG was associated with higher SFA, n-6 PUFA, and LA levels. These results are also consistent with our findings. Furthermore, Brett et al., [32] reported that mothers with obesity were more likely to have impaired transfer of DHA to their fetus during pregnancy.

Meher et al. [33] observed lower levels of n-3 PUFAs (1.90 ± 0.67%) in maternal erythrocytes and higher levels n-6 PUFAs (38.78 ± 5.01%) and SFAs (34.55±3.09%) at delivery in women delivering normal-birth-weight babies, compared with our findings, in which the maternal plasma total n-3 PUFA levels were 1.5 ± 0.6%, the total n-6 PUFA levels were 18.6 ± 6.1%, and the total SFA levels were 26.3 ± 4.4% at delivery. In the above previous study, DHA reached levels of 1.09 ± 0.44%, whereas AA reached levels of 6.22 ± 3.02% at delivery in women delivering normal-birth-weight babies. These findings are also comparable to our results, wherein the DHA levels were 0.78 ± 0.4% and the AA levels were 4.5 ± 1.3%. Moreover, the importance of n-3 PUFAs in fetal development has been documented [34, 35].

Significant linear correlations have been found between maternal and fetal levels of n-3 and n-6 PUFAs, and parallel increases in plasma DHA levels were observed in the mothers and newborns after fish-oil supplementation during pregnancy [36,37]. It has been shown that n-3 and n-6 PUFAs compete for desaturases; hence, an excess of certain FAs may impair the availability of others [38]. During the last trimester of pregnancy, fetal requirements for AA and DHA are especially high because of the rapid synthesis of brain tissue; accordingly, pregnancy may deplete maternal DHA stores, resulting in a suboptimal DHA supply to the fetus [39].

This study had a few limitations. Our results were based on cross-sectional data and thus need to be verified by longitudinal studies. Moreover, the study sample was small and taken from a single center. These factors might limit the generalization of our findings. Further studies are required to explore the causality of the associations between pre-pregnancy BMI, GWG, and plasma FA levels, preferably studies with longitudinal measurements of both weight and FA levels before and during pregnancy.

## Conclusions:

It is of interest to examine the association between pre-pregnancy BMI, GWG, and plasma FA profiles in pregnant women. Our results showed that a higher pre-pregnancy BMI and GWG were associated with lipid profiles in Saudi women after giving birth. We believe that future studies to examine these associations are warranted. Controlling the levels of long-chain PUFAs during pregnancy could be beneficial to the prevention of obesity and GWG. It is worth considering conducting trials that investigate the effects of n-3 PUFA supplementation on birth outcomes in women with a diet that is very low in n- n-3 PUFAs, especially women with obesity.

## Figures and Tables

**Table 1:** Characteristics of participants according to body mass index category

Characteristic	Total group (238)	Normal weight, n = 118 (49.6%)	Overweight, n = 86 (36.1%)	Obesity, n = 34 (14.3%)	P-value
Age (years)	29.2 ± 5.1	28.1 ± 5.2	30.3 ± 4.8	30.5 ± 4.8	0.00*
Education level, N (%)					
Primary school	11 (4.7%)	6 (5%)	3 (3.5%)	2 (5.9%)	0.04*
Secondary school	22 (9.2%)	5 (4.2%)	10 (11.6%)	7 (20.6%)	
High school	82 (34.5%)	38 (32.2%)	33 (38.4%)	11 (32.4%)	
Bachelor's degree	123 (51.7%)	69 (58.5%)	40 (46.5%)	14 (41.2%)	
Employment status, N (%)					
Employed	49 (20.6%)	26 (22%)	14 (16.3%)	9 (26.5%)	0.39
Unemployed	189 (79.4%)	92 (78%)	72 (83.7%)	25 (73.5%)	
Income (Saudi riyal), N (%)					
>6000	66 (22.7%)	35 (29.7%)	19 (22.1%)	12 (35.3%)	0.37
6000–10,000	100 (42%)	51 (43.2%)	39 (45.3%)	10 (29.4%)	
<10,000	72 (30.3%)	32 (27.1%)	12 (32.6%)	12 (35.3%)	
Delivery method					
Vaginal	150 (63%)	97 (82.2%)	46 (53.5%)	7 (20.6%)	0.00*
Cesarean	88 (37%)	21 (17.8%)	40 (46.5%)	27 (79.4%)	
Parity					
0	49 (20.6%)	29 (24.6%)	15 (17.4%)	5 (14.7%)	0.02*
1	90 (37.8%)	49 (41.5%)	34 (39.5%)	7 (20.6%)	
>2	99 (41.6%)	40 (33.9%)	37 (43%)	22 (64.7%)	
Pre-pregnancy BMI (kg/m2)	26.4 ± 5.1	22.4 ± 2.9	28.9 ± 2.3	33.9 ± 4.1	0.000**
Gestational weight gain (kg)	10.7 ± 5.2	11.5 ± 4.2	10.7 ± 4.6	15.2 ± 7.1	0.000**
Inadequate, N (%)	68 (28.6%)	65 (55.1%)	3 (3.5%)	0	0.000**
Adequate, N (%)	102 (42.9%)	47 (39.8%)	48 (55.8%)	7 (20.6%)	
Excessive, N (%)	68 (28.6%)	6 (5.1%)	35 (40.7%)	27 (79.4%)	
Gestational age at delivery (weeks)	38.8 ± 1.2	38.8 ± 1.2	38.8 ± 1.1	38.7 ± 1.4	0.99
*P < 0.05, **P < 0.001. BMI, body mass index

**Table 2:** Maternal plasma fatty acid profiles according to body mass index category

Fatty acids (weight %)	Entire groupa -238	Normal weight, n = 118 (49.6%)	Overweight, n = 86 (36.1%)	Obesity, n = 34 (14.3%)	P-value
Total SFAs	26.3 ± 4.4	26.5 ± 4.1	26.3 ± 4.7	27.4 ± 4.6	0.04*
Total MUFAs	18.8 ± 4.1	18.3 ± 4.3	18.6 ± 4.4	19.2 ± 3.7	0.05*
n-3 PUFAs					
Total n-3 PUFAs	1.5 ± 0.6	1.8 ± 0.7	1.6 ± 0.6	1.4 ± 0.6	0.61
ALA	0.77 ± 0.6	0.92 ± 0.7	0.79 ± 0.6	0.69 ± 0.5	0.18
DHA	0.78 ± 0.4	0.81 ± 0.3	0.81 ± 0.4	0.67 ± 0.3	0.19
n-6 PUFAs					
Total n-6 PUFAs	18.6 ± 6.1	16.9 ± 6.3	19.5 ± 6.4	21.8 ± 5.2	0.000**
LA	12.7 ± 4.7	11.2 ± 4.7	13.5 ± 4.5	15.7 ± 3.2	0.000**
DGLA	1.4 ± 0.4	1.4 ± 0.4	1.4 ± 0.5	1.4 ± 0.5	0.93
AA	4.5 ± 1.3	4.3 ± 1.2	4.6 ± 1.4	4.7 ± 1.5	0.17
n-6 to n-3 PUFA ratio	12.4 ± 6.3	9.4 ± 6.7	13.5 ± 5.2	15.5 ± 5.4	0.01*
aData are expressed as means ± standard deviation. SFAs include 14:0, 16:0, and 18:0; MUFAs include 16:1 n-7 and 18:1 n-9; n-3 PUFAs include 18:3 n-3 and 22:6 n-3; and n-6 PUFAs include 18:2 n-6, 20:3 n-6, and 20:4 n-6. *P < 0.05, **P < 0.001. SFAs, saturated fatty acids; MUFAs, monounsaturated fatty acids; PUFAs, polyunsaturated fatty acids; ALA, α-linolenic acid; DHA, docosahexaenoic acid; LA, linoleic acid; DGLA, dihomo-γ-linolenic acid; AA, arachidonic acid.

**Table 3:** Associations of maternal body mass index and gestational weight gain with fatty acids

Fatty acids	Body mass index		Gestational weight gain	
	Overweighta mean (95% CI), N = 86	Obesitya mean (95% CI), N = 34	Inadequatea mean (95% CI), N = 68	Excessivea mean (95% CI), N = 68
Total SFAs	0.88 (0.78-1)	1.01 (0.84-1.29)*	0.92 (0.81-1.05)	2.02 (0.81-3.07)*
Total MUFAs	1.03 (0.91-1.15)	0.83 (0.68-1.1)*	1.13 (0.98-1.29)	0.97 (0.84-1.15)
Total n-3 PUFAs	-0.77 (0.49-1.2)	-0.91 (0.49-1.68)	0.74 (0.47-1.19)	0.65 (0.39-1.08)
ALA	-0.75 (0.34-1.67)	-1.04 (0.32-1.34)	0.44 (0.17-1.11)	0.41 (0.13-1.28)
DHA	-0.67 (0.21-2.13)	-0.21 (0.03-1.19)	0.32 (0.08-1.18)	0.31 (0.07-1.42)
Total n-6 PUFAs	1.101 (1.04-1.16)**	1.21 (1.11-1.31)***	0.98 (0.92-1.03)	1.06 (1-1.12)*
LA	1.12 (1.02-1.24)*	1.37 (1.16-1.63)***	0.95 (0.92-1.11)	1.13 (0.91-1.43)*
DGLA	0.75 (0.31-1.87)	0.73 (0.21-1.57)	0.51 (0.19-1.17)	0.77 (0.29-2.04)
AA	1.81 (0.28-2.48)	1.96 (0.98-2.85)	1.2 (0.99-1.73)	1.33 (0.26-1.86)
n-6 to n-3 PUFA ratio	0.98 (0.89-1.1)	0.96 (0.82-1.12)	0.93 (0.98-1.04)	0.92 (081-1.06)
aFatty acid levels are expressed as percentages of total fatty acids and represented as geometric means with 95% confidence intervals. *P < 0.05, **P < 0.001, ***P < 0.0001. SFAs, saturated fatty acids; MUFAs, monounsaturated fatty acids; PUFAs, polyunsaturated fatty acids; ALA, α-linolenic acid; DHA, docosahexaenoic acid; LA, linoleic acid; DGLA, dihomo-γ-linolenic acid; AA, arachidonic acid.
